# Supplementary material for: Asymmetric Synthesis of Quaternary Hydantoins via a Palladium-Catalyzed Aza-Heck Cyclization
Source: J Am Chem Soc. 2025 Nov 14;147(49):44692–8. doi: 10.1021/jacs.5c16022 (PMC12703750; doi:10.1021/jacs.5c16022)
Supplement: Supplementary file 2 [file ja5c16022_si_002.zip › All NMR FID Files/8/8_CarbonNMR/pdata/1/email_TDI01-064 xterize CAPT_1_1.pdf]

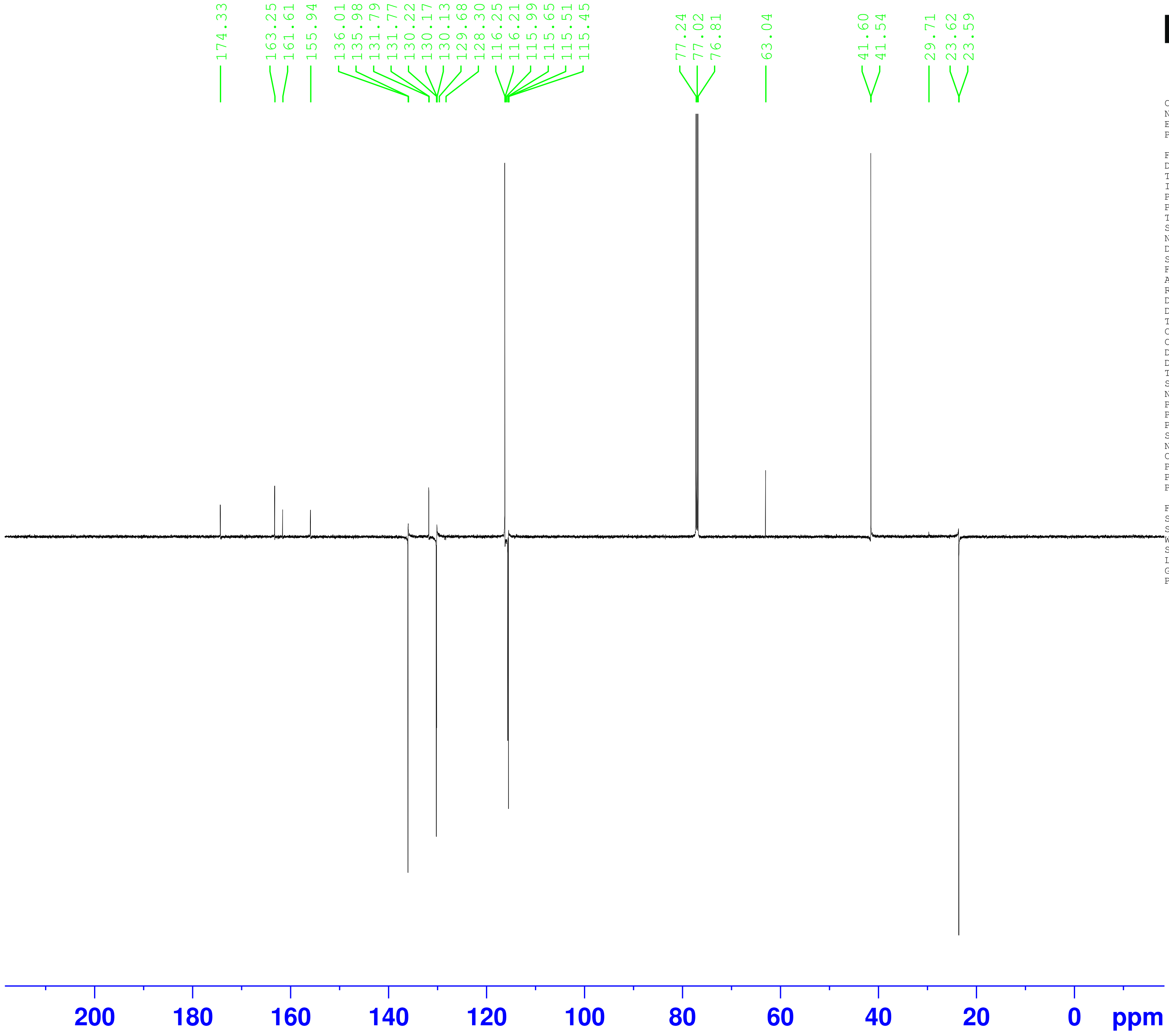


```
Current Data Parameters
NAME     TDI01-064 xterize CAPT
EXPNO                 1
PROCNO                1

F2 - Acquisition Parameters
Date_          20250220
Time               5.48 h
INSTRUM  Avance Neo 600 at University of Delaware
PROBHD   Z185170_0001 (
PULPROG            jmod
TD                65536
SOLVENT           CDCl3
NS                 2048
DS                    4
SWH           35714.286 Hz
FIDRES         1.089913 Hz
AQ            0.9175040 sec
RG                  101
DW               14.000 usec
DE                19.25 usec
TE                298.0 K
CNST2       145.0000000
CNST11        1.0000000
D1           2.00000000 sec
D20          0.00689655 sec
TD0                   1
SFO1        150.9178988 MHz
NUC1                13C
P1                11.98 usec
P2                23.96 usec
PLW1       106.00000000 W
SFO2        600.1324005 MHz
NUC2                 1H
CPDPRG[2         waltz65
PCPD2             70.00 usec
PLW2         6.90000010 W
PLW12        0.09170600 W

F2 - Processing parameters
SI                32768
SF          150.9028085 MHz
WDW                  EM
SSB                   0
LB                 1.00 Hz
GB                    0
PC                 1.40
```
